# Supplementary material for: Development of a Deep Learning Model for Malignant Small Bowel Tumors Survival: A SEER-Based Study
Source: Diagnostics (Basel). 2022 May 17;12(5):1247. doi: 10.3390/diagnostics12051247 (PMC9141623; doi:10.3390/diagnostics12051247)
Supplement: Supplementary file 1 [file diagnostics-12-01247-s001.zip › diagnostics-1707093-supplementary.pdf]

diagnostics

MDPI

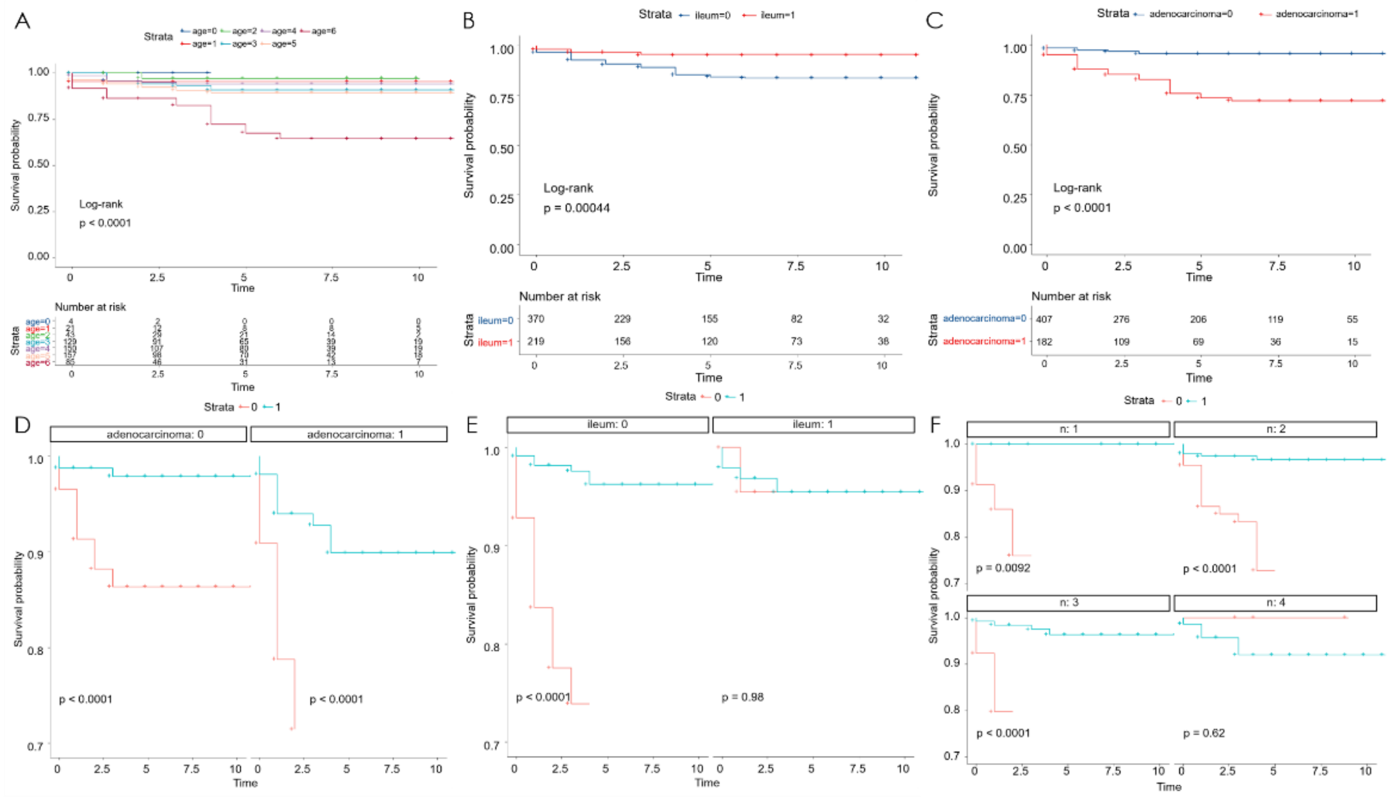

Figure S1. K–M analysis of independent risk factors for cancer-specific survival. (**A**). Age; (**B**). primary tumor site (ileum); (**C**). histological type (adenocarcinoma); (**D**–**F**). the efficacy of surgery on prognosis in the sub-groups of patients who had adenocarcinoma (**D**), had tumor located in ileum (**E**) and had N2 stage disease (**F**). (**A**) Age = 0 means age < 30, age = 1 means 30 < age ≤ 40, age = 2 means 40 < age ≤ 50, age = 3 means 50 < age ≤ 60, age = 4 means 60 < age ≤ 70, age = 5 means 70 < age ≤ 80, age = 6 means age ≥ 80. (**D**–**F**) Strata = 0 means no surgery, strata = 1 means surgery, *n*:1 means Nx, *n*:2 means N0, *n*:3 means N1, *n*:4 means N2.

Table S1. C-indices of Cox-PH and DeepSurv models based on different hyperparameters.

Model	Reference	C-index	Layers	Nodes/Layer	Dropout	Learning Rate (LR)
Cox-PH	-	0.866	-	-	-	-
DeepSurv	Adeoye et al. [1]	0.863	4	32, 64, 128, 256	0.3	0.009545
		**0.871**	4	32, 64, 128, 256	0.2	0.020092
		0.849	4	32, 64, 128, 256	0.1	0.073907
	Katzman et al. [2]	0.860	2	48, 48	0.147	0.089022
		0.845	3	17, 17, 17	0.401	0.155568
		0.838	1	41	0.160	0.061359
		0.848	1	45	0.109	0.061359
		0.842	1	8	0.661	0.155568

References

1. Adeoye, J.; Koohi-Moghadam, M.; Lo, A.W.I.; Tsang, R.K.-Y.; Chow, V.L.Y.; Zheng, L.-W.; Choi, S.-W.; Thomson, P.; Su, Y.-X. Deep Learning Predicts the Malignant-Transformation-Free Survival of Oral Potentially Malignant Disorders. *Cancers* **2021**, *13*, 6054, https://doi.org/10.3390/cancers13236054.
2. Katzman, J.L.; Shaham, U.; Cloninger, A.; Bates, J.; Jiang, T.; Kluger, Y. DeepSurv: Personalized treatment recommender system using a Cox proportional hazards deep neural network. *BMC Med Res. Methodol.* **2018**, *18*, 24, https://doi.org/10.1186/s12874-018-0482-1.
